# The Effect of Medical Cannabis on Pain Level and Quality of Sleep among Rheumatology Clinic Outpatients

**DOI:** 10.1155/2021/1756588

**Published:** 2021-09-06

**Authors:** George Habib, Fadi Khazin, Suheil Artul

**Affiliations:** ^1^Rheumatology Unit, Laniado Hospital, Netanya, Israel; ^2^Rheumatology Clinic, Nazareth Hospital, Nazareth, Israel; ^3^Faculty of Medicine, Azrieli Faculty of Medicine, Bar-Ilan University, Safed, Israel; ^4^Orthopedic Department, Lady Davis Medical Center, Haifa, Israel; ^5^Department of Radiology, Nazareth Hospital, Nazareth, Israel

## Abstract

**Introduction:**

Medical cannabis (MC) is becoming increasingly popular for the treatment of chronic pain conditions. In this study, we evaluated the effect of MC treatment on pain level and quality of sleep of patients with different medical conditions at the rheumatology clinic.

**Methods:**

Patients licensed for the use of MC at the rheumatology clinics at different settings were located and contacted. Their demographic and clinical parameters were documented, including type of medical cannabis consumed, way of consumption, and current monthly consumed amount. These patients were contacted by phone and asked about the effect on pain level and quality of sleep.

**Results:**

A total of 351 patients were located, and 319 completed the questionnaire. Mean age was 46 ± 12 years, 76% were female, 82% had fibromyalgia, ∼9% had mechanical problems, ∼4% had inflammatory problems, ∼4% had neurological problems, and ∼1% had other problems. The average monthly consumed dose of MC was 31, 35, 36, and 32 g, with mean pain level reduction of 77%, 82%, 83%, and 57%, and mean sleep quality improvement of 78%, 71%, 87%, and 76% among patients with fibromyalgia, mechanical, neuropathic, and inflammatory problems, respectively. Mean THC and CBD contents were 18.38% ± 4.96 and 2.62% ± 4.87, respectively. The THC concentration, duration of MC consumption, and MC consumption dose had independent significant correlations with pain reduction while only the duration of MC consumption had an independent significant correlation with sleep quality improvement.

**Conclusions:**

MC had a favorable effect on pain level and quality of sleep among all spectrums of problems at the rheumatology clinic.

## 1. Introduction

Medical cannabis (MC) is gaining increasing popularity as a treatment for severe cases of chronic pain syndromes, including musculoskeletal and neuropathic conditions [1]. It has been shown that MC is very helpful for fibromyalgia patients [2–4]. Its wide range of favorable effects on pain, sleep problems, muscle spasms, anxiety, depression, and fatigue makes it very appealing for fibromyalgia patients.

Currently, despite the introduction of biological treatment for different inflammatory rheumatic diseases, including rheumatoid arthritis, psoriatic arthritis, and ankylosing spondylitis, a small proportion of the patients with these conditions either do not or only partially respond to the biological treatment, with their ongoing pain affecting their sleep. These biological treatments are associated with increased risk of infection, including herpes zoster and/or tuberculosis and even increased incidence of malignancy [5]. Some patients also develop secondary fibromyalgia [6]. Such patients may benefit from cannabis treatment.

With the expanding nonsurgical treatments of degenerative musculoskeletal disease, rheumatologists are also dealing more and more with patients with such ailments at the rheumatology clinic. Degenerative diseases are the most common musculoskeletal problems, where painkillers of different strengths are the main treatment option. Some people with such problems also consume narcotics [7]. Recently, with the American opioid crisis, the huge number of strong opioid consumers and the number of related fatalities have been shocking [8]. Thus, an increasing number of patients with mechanical problems are seeking cannabis for pain relief [1]. MC has also shown promising results in the treatment of resistant neuropathic problems, including neuropathic pain and tremor [9–11].

The adverse effects of MC have been reported to be mostly mild and tolerable, and some have been reported to be transient, with a relatively low dependency rate or tolerance [12]. Such adverse effects include weakness, dizziness, nausea, headache, abdominal pain, palpitations, mouth bitterness, dry eyes, dry mouth, and/or diarrhea.

Cannabis is still considered a prohibited drug in Israel, and a license is issued for its use by designated medical doctors or by the Israeli MC Agency (IMCA). It is indicated for a wide variety of clinical issues but mainly for malignancy-associated symptoms/signs, resistant chronic pain syndromes, resistant neurologic problems, and resistant mechanical problems.

Here, we summarize all the patients registered at rheumatology clinics who were treated with MC to prospectively evaluate the effect of MC on their pain level and quality of sleep.

## 2. Methods

All the patients who had been treated with MC in the registry of two outpatient rheumatology clinics, Laniado Hospital in Netanya and Nazareth Hospital in Nazareth, in addition to those in the registries of the rheumatology clinics of four health insurance services in Israel (i.e., Maccabi, Clalit, Leumit, and Meuhedet) followed by the first author, were located (a total of six outpatient clinics in northern Israel). About 450 patient visits are seen monthly in these clinics. Only patients who used MC for at least two months prior to the MC reform in May 2019 were included in the study. The reason behind this is that prior to MC reform, patients were able to change the type of MC they consumed within few weeks if the cannabis was not beneficial and also had free guidance and assistance in how to use MC. Following the reform, patients had no free guidance and support and took many months for them to be able to change the type of cannabis if these types were not helpful, especially the new users of MC. Therefore, we wanted to guarantee that all the patients in our study had the chance to change their type of cannabis at least once, once their initial cannabis used did not suit them, and also had guidance on how to use MC.

The study started nearly 7 months prior to submitting the manuscript.

These patients were contacted by phone and were asked if they wanted to participate in this study. After they gave their consent (by phone), their demographic and clinical parameters were documented, including their age, gender, indication for MC license acquisition, the current monthly amount of cannabis consumption, current THC and CBD concentrations, way of MC consumption, and MC impact on pain and sleep quality. The impact on pain was evaluated through a standard questionnaire item: “Grade the effect of your current MC consumption amount on your pain level from 0 to 100, where 0 stands for no change and 100 stands for full improvement.” As for the impact on sleep quality, it was evaluated through the following standard questionnaire item: “Grade the effect of your current MC consumption amount on the quality of your sleep from 0 to 100, where 0 stands for no change and 100 stands for full improvement or normal refreshing sleep.”

A linear regression analysis using SPSS software, version 22, assessed the correlation between different parameters and the percentage of improvement of either pain or quality of sleep.

This study was approved by the local Ethics Committee of the Nazareth Hospital.

## 3. Results

A total of 351 patients were identified; 339 were contacted; 10 had stopped MC consumption earlier (9 with fibromyalgia and 1 with an inflammatory problem); and 319 provided a complete set of data. The first patient was documented in the registry nearly 6 years prior to the study.

Table 1 summarizes the indications of MC license acquisition, age, gender, duration of MC consumption, and duration of disease for which the patient is licensed to use MC. Of the patients enrolled in the study, 260 (82%) had fibromyalgia, 14 (4.4%) had mechanical back pain, 8 had physical injury, 7 had rheumatoid arthritis, and 7 had diabetic neuropathy. The rest had different degenerative, inflammatory, neuropathic, or other entities. The main indications in all of the patients were pain, except for 4 patients; 2 for essential tremor and the other 2 with Parkinson's and treated with MC mainly for tremor and spasticity.

Table 2 summarizes the different parameters regarding MC consumption that were considered in this study: monthly dose, way of consumption, and THC and CBD contents. The average monthly dose in all the groups was 31−36 g (MC was supplied in bags of 10 g each), and the mean percentages of THC and CBD were 18.38 ± 4.96 (range: 9.3–27) and 2.62 ± 4.87 (range: 0.1–13.9), respectively. The percentages of the patients in the different groups who consumed MC only by smoking/vaping were 62−82%.

Table 3 summarizes the impact of MC consumption on pain level and sleep quality.

The mean pain improvement in the different groups was 57−83%, and the mean improvement in sleep quality was 71−87%.

Table 4 summarizes the results of the multivariate regression analysis that was performed to determine which different parameters considered in the study significantly correlated with the percentage of improvement in pain level and sleep quality. The THC concentration dose and the duration of MC treatment significantly correlated with the level of pain response, and only MC dose significantly correlated with sleep quality improvement.

## 4. Discussion

Most of the patients in the clinics that had been approved for MC treatment were fibromyalgia patients (82%), which is not surprising because fibromyalgia is a relatively common syndrome, and the number of patients with such ailment among all the patients at the rheumatology clinics is relatively high [13]. In addition, many patients with fibromyalgia do not have an effective treatment, and not few of them had experienced serious adverse effects prior to MC approval, from these treatments [14, 15]. The spectrum of medical treatments used by fibromyalgia patients included simple analgesics, tricyclic antidepressants, tramadol, duloxetine, pregabalin, nonsteroidal anti-inflammatory meds, benzodiazepines, strong opioids, selective serotonin reuptake inhibitors, and/or others. In addition, fibromyalgia patients have multiple symptoms and signs, including muscle spasms, peripheral numbness, anxiety, depression, and insomnia. MC can be of great help for all these symptoms/signs [16, 17]. All these factors make MC treatment very appealing for fibromyalgia patients.

The average improvement in sleep quality and pain level among the fibromyalgia patients in this study was relatively high: 78 and 77%, respectively, similar to the results of other studies. In a prospective study by Sagy et al. [3], 81.1% of the patients achieved treatment success defined by moderate improvement in their condition while still receiving MC, without experiencing serious adverse events. Of these patients, 67.3% used flowers, and 12% used a combination of oil and flowers. Ninety percent of those who had a high level of pain at baseline (8−10 on a scale from 0 to 10) reported improvement in pain intensity, with a median of 5 after 6 months of follow-up. In fact, none had shown deterioration, and a third experienced mild adverse effects not resulting in treatment modification.

In another prospective study by Giorgi et al. [4], THC-rich (22%) and balanced THC + CBD (6.3 + 8%, respectively) extracts were added to MC for fibromyalgia patients under the standard analgesic treatment. Of these patients, 44 and 33% showed significant improvement in the Pittsburgh Sleep Quality Index and the Fibromyalgia Impact Questionnaire, respectively.

The leading theory behind the development of fibromyalgia is central sensitization, with dysregulation of pain perception and hyperalgesia [18], and the mechanism through which MC alleviates the symptoms of fibromyalgia is not yet fully understood. The brain is rich in CB1 receptors, including brain areas that are relevant to fibromyalgia, such as the cortex (for assessing pain), the hypothalamus (important in sleep), the hippocampus (for the memory), and the basal ganglia and cerebellum (for movement) [19]. In addition, the role of the CB2 receptors (which are mostly peripherally distributed) in muscle and tendon pain relief in fibromyalgia following cannabis treatment is not clear. There are nearly no reports about the existence of CB2 receptors in the muscles or tendons [20]. One of the theories behind the pathogenesis of fibromyalgia, however, is endocannabinoid deficiency. It has been shown that patients with migraines have low cerebrospinal fluid levels of amantadine and patients with posttrauma stress syndrome have imaging evidence of endocannabinoid hypofunction [21].

About 4% of the MC-licensed patients in our registry had inflammatory problems. The impact of MC on such patients' pain level was much less favorable than that on their sleep quality (57 vs. 76%). These patients (with inflammatory problems) also had higher CBD contents than the other patients. Higher doses of CBD specifically might be useful for sleep concerns [22].

It has been shown that CBD, in particular, has an anti-inflammatory effect [23]. Cannabis in general has immunologic effects at both the cellular and cytokine levels. It has resulted in a decrease in the number of inflammatory cells at the inflamed joints and in the downregulation of TNF, IL-6, IL-1, IL-17, IL-23, and other cytokine levels [24]. Many of the patients in this study with inflammatory problems, however, were still having synovitis clinically under MC treatment, and it seems that the favorable effect of MC in these patients was mainly the antipain effect rather than the anti-inflammatory effect. With regard to the C-reactive protein (C-RP) levels under cannabis treatment, there was some decrease in them, but they did not reach the normal levels (data not shown).

Among the patients in this study, 9% had mechanical problems. The mean improvement in their pain level and sleep quality was also relatively high: 82 and 71%, respectively. A wide range of cannabinoid receptors was expressed on the chondrocytes of the patients with osteoarthritis of the knee. These receptors included CB1, CB2, GPR55, PPAR*α*, and PPAR*γ* [25]. The level of expression did not change with the level of osteoarthritis changes. CB1 and CB2 protein and RNA were also present in the synovia of the OA patients. In addition, both anandamide endocannabinoids (AEA) and 2-arachidonoylglycerol (2-AG) were identified in the synovial fluid of these patients but not from the synovial fluid of the normal volunteers [26]. Adding MC to the patients with low back pain and fibromyalgia resulted in a significant alleviation of their back pain [27].

The highest figures of pain level and quality of sleep improvement were observed among the patients with neuropathic problems. Most of the patients had peripheral neuropathy, with pain and paraesthesia being the main distressing and annoying factors. The other patients had radicular pain, and one patient had a severe tremor. Cannabis had been shown to significantly reduce and improve neuropathic pain both in animals and humans [28]. One study had shown that CBD induces analgesia in an animal model, predominantly through TRPV1 activation [29]. Cannabis had also been shown to have a preventive effect on chemotherapy-induced neuropathic pain [30]. Other than the aforementioned major categories, it can be seen that MC cannabis was used by patients with the complex regional pain syndrome, peripheral vascular disease, and osteochondroma with favorable effects.

All the patients in this study had high THC levels with relatively low CBD concentrations. THC is known to be a powerful component for pain relief and to be more potent than CBD. The highest CBD content was seen among the patients with inflammatory arthritis. As mentioned earlier, CBD has an anti-inflammatory effect, but this effect seemed to be a weak one among the patients in this study.

The monthly doses that were documented did not necessarily reflect the real amounts needed by the patients to control their symptoms. The requested dose of MC needed to be approved by the IMCA, and there was a high percentage of refusal. In general, the fibromyalgia patients reported that 1 g per day was the average dose that they needed, while among the patients with mechanical and neurologic problems, there was a demand for higher doses of MC.

In the multivariate regression analysis of all the patients in this study, the THC concentration, cannabis dose, and duration of cannabis treatment were found to be significantly correlated with pain improvement, and the duration of cannabis treatment was found to be significantly correlated with sleep quality improvement. These results can be explained by the known fact that THC is an important cannabinoid for pain relief and is much more powerful than CBD [31, 32]. The duration of cannabis treatment is important in acquiring experience in using cannabis and also in trying out different species and ways of consumption to ultimately get to the right species with the best benefit to the patient. Demanding for and getting higher doses of cannabis do not necessarily represent cannabis tolerance or failure, but in many patients in this study, it reflected high satisfaction and desire for zero pain during a 24-hour time. In addition, many of the patients gained high expertise in the effects of different species of cannabis (different products with similar THC and CBD concentrations and/or different products with different THC and CBD concentrations) and tried acquiring many species at the same time to enjoy the different effects at different times of the day.

The lack of correlation between improvement in quality of sleep and CBD concentration could be related to the low doses of CBD relatively used by the patient (Table 2). Higher doses of CBD were widely available following the MC reform in Israel. A recent study in animals had found that THC, rather than CBD, was more effective in promoting NREM sleep [33].

The patients who stopped taking medical cannabis earlier (ten patients) were not included in the study; seven of them did so due to insufficient or lack of effect. The other two patients did so due to adverse effects; severe bitterness of the mouth due to the consumption of extract in the first; and severe diarrhea accompanied by abdominal pain in the second. The last patients stopped MC treatment due to cost issues.

This study had several limitations. One of these was the lack of documentation of adverse effects and concomitant medications. The data from the literature are quite consistent with the rates of adverse effects in 25−30% of the patients using medical cannabis in this study (mostly fibromyalgia patients), but the adverse effects were mostly mild to moderate, not leading to discontinuation of MC. Yet two patients on oil from the study cohort stopped MC consumption due to intolerable adverse effects.

Regarding concomitant treatment, one of the requirements of the IMCA for approving MC dose rising is that the patients are still on painkillers or other specific medications for their primary disease, in order to justify MC dose raising. Thus, patients asking for MC dose rising still needed to buy their medications from the pharmacy even if these meds were ineffective and/or produced some adverse effects. Thus, concomitant treatments besides MC did not really reflect the need for them by MC-consuming patients in our study and could not reflect the real benefits that patients in our study had obtained from MC treatment.

The patients in this study evidently did not represent the real-world MC treatment prescribed by authorized physicians without the need for approval by a higher authority and without restrictions on the doses to be taken by the patients.

## 5. Conclusions

MC has a favorable effect on pain level and sleep quality among nearly the entire spectrum of resistant “chronic pain syndromes” seen or referred to rheumatology clinics, including inflammatory diseases resistant to biological treatment, although the effect of MC on synovitis was relatively mild.

Cannabis should be seriously considered in every “chronic pain condition” whenever the accepted modalities of treatment are insufficient for alleviating patient's pain and sleep problems.

## Figures and Tables

**Table 1:** Rheumatologic diagnoses and demographic characteristics of 319 patients receiving medical cannabis different rheumatology outpatient clinics in North of Israel.

Type of disease	Number (%)	Age^*∗*^	Gender F:M	Duration of MC use (years)	Duration of disease (years)
Fibromyalgia	260 (81.5)	45 ± 11.8	217:43	2.89 ± 2.1	5.6 ± 3.9
Mechanical back pain	14 (4.4)	59 ± 10.3	6:8	1.9 ± 1.4	10.5 ± 5.4
Physical injury	8 (2.5)	36.6 ± 12.3	2:6	3.2 ± 2.3	7.1 ± 5.2
Rheumatoid arthritis	7 2.2)	41.6 ± 6.5	5:2	1.5 ± 0.85	7.37 ± 4.24
Diabetic neuropathy	7 (2.2)	52.6 ± 10.5	2:5	2.87 ± 1.9	11.86 ± 7.36
Psoriatic arthritis	3 (0.9)	46.3 ± 8.7	1:2	3 ± 1	8.3 ± 2.1
Sacroiliitis	3 (0.9)	35.7 ± 7.5	2:1	2.33 ± 0.58	9.2 ± 3.6
CRPS	3 (0.9)	32.3 ± 5.5	0:3	4.33 ± 1.76	7.2 ± 3.4
Knee osteoarthritis	2 (0.6)	69 ± 4.2	2:0	2.25 ± 1.1	16.2 ± 8.5
FMF	2 (0.6)	31 ± 2.8	0:2	3.25 ± 1.34	19 ± 4.24
Tremor	2 (0.6)	58 ± 19.8	2:0	3.75 ± 3.18	8.28
Osteoporotic fractures	2 (0.6)	69 ± 4.2	2:0	3.25 ± 1.1	11.5 ± 5.6
Spinal stenosis	2 (0.6	59 ± 4.3	1:1	3.5 ± 1.4	8 ± 2.83
Parkinson	2 (0.6)	53 ± 6.4	1:1	2.4 ± 2.26	16.5 ± 7.8
Osteochondroma	1 (0.3)	25	0:1	3.5	12
PVD	1 (0.3)	65	0:1	2.75	18

^*∗*^Mean ± standard deviation. CRPS = complex regional pain syndrome, FMF = familial Mediterranean fever, PVD = peripheral vascular disease, MC = medical cannabis, and F:M = female:male.

**Table 2:** Characteristics of MC use and THC and CBD contents among the different groups of patients.

Parameter	Fibromyalgia	Degenerative problems	Inflammatory problems	Neuropathic pain
THC^*∗*^ (%)	9.3–27	14–24	18–22	16–24
CBD^*∗*^ (%)	0.1–13.9	<1	3–10	<3
Monthly amount	31	35	32	36
Smoking/vaping (%)	78	74	62	82
Oil alone (%)	13	22	28	9
Combination (%)	9	4	10	4
MC stopped (%)	5	0	6	0

^*∗*^Range, mean. Inflammatory problems included rheumatoid arthritis, psoriatic arthritis, FMF, and sacroiliitis. Degenerative problems included mechanical back pain, knee osteoarthritis, physical injury, and spinal stenosis. Neuropathic problems included diabetic neuropathy, Parkinson's, and tremor.

**Table 3:** Pain and quality of sleep mean response to MC treatment.

Type	Fibromyalgia (%)	Degenerative problems (%)	Inflammatory problems (%)	Neuropathic pain (%)
Pain level	77^*∗*^	82^*∗*^	57^*∗*^	83^*∗*^
Sleep quality	78^*∗*^	71^*∗*^	76^*∗*^	87^*∗*^

MC = medical cannabis. ^*∗*^Current mean improvement in pain and sleep compared to the period just prior to medical cannabis use. Inflammatory problems included rheumatoid arthritis, psoriatic arthritis, FMF, and sacroiliitis. Degenerative problems included mechanical back pain, knee osteoarthritis, physical injury, and spinal stenosis. Neuropathic problems included diabetic neuropathy, Parkinson's, and tremor.

**Table 4:** Multilinear regression analysis correlating pain and sleep response (separately) with different independent parameters.

Parameter	Pain *P* value	Sleep *P* value
Gender	0.23	0.46
Age	0.46	0.78
Category		
Fibromyalgia	0.54	0.38
Neuropathic	0.36	0.65
Degenerative	0.47	0.544
Inflammatory	0.87	0.16
Cannabis dose	0.016^*∗*^	0.08
Duration of MC use	0.032^*∗*^	0.034^*∗*^
Duration of illness	0.520	0.26
THC concentration	0.023^*∗*^	0.18
CBD concentration	0.16	0.23

^*∗*^Significant value.

## Data Availability

The data used to support the findings of this study are available from the corresponding author upon request.
